# Seed-Derived Microbial Colonization of Wild Emmer and Domesticated Bread Wheat (*Triticum dicoccoides* and *T. aestivum*) Seedlings Shows Pronounced Differences in Overall Diversity and Composition

**DOI:** 10.1128/mBio.02637-20

**Published:** 2020-11-17

**Authors:** Ezgi Özkurt, M. Amine Hassani, Uğur Sesiz, Sven Künzel, Tal Dagan, Hakan Özkan, Eva H. Stukenbrock

**Affiliations:** a Environmental Genomics, Christian-Albrechts University of Kiel, Kiel, Germany; b Environmental Genomics, Max Planck Institute for Evolutionary Biology, Plön, Germany; c Department of Field Crops, Faculty of Agriculture, University of Çukurova, Adana, Turkey; d Evolutionary Genetics, Max Planck Institute for Evolutionary Biology, Plön, Germany; e Institute of General Microbiology, Christian-Albrechts University of Kiel, Kiel, Germany; Universidad de Córdoba

**Keywords:** seed-associated microbiome, plant domestication, plant breeding, microbiota assembly, agriculture, plant microbiota, seed microbiota, wheat domestication, wheat microbiota

## Abstract

The composition of the plant microbiota may be altered by ecological and evolutionary changes in the host population. Seed-associated microbiota, expected to be largely vertically transferred, have the potential to coadapt with their host over generations. Strong directional selection and changes in the genetic composition of plants during domestication and cultivation may have impacted the assembly and transmission of seed-associated microbiota. Nonetheless, the effect of plant speciation and domestication on the composition of these microbes is poorly understood. Here, we have investigated the composition of bacteria and fungi associated with the wild emmer wheat (Triticum dicoccoides) and domesticated bread wheat (Triticum aestivum). We show that vertically transmitted bacteria, but not fungi, of domesticated bread wheat species *T. aestivum* are less diverse and more inconsistent among individual plants compared to those of the wild emmer wheat species *T. dicoccoides.* We propagated wheat seeds under sterile conditions to characterize the colonization of seedlings by seed-associated microbes. Hereby, we show markedly different community compositions and diversities of leaf and root colonizers of the domesticated bread wheat compared to the wild emmer wheat. By propagating the wild emmer wheat and domesticated bread wheat in two different soils, we furthermore reveal a small effect of plant genotype on microbiota assembly. Our results suggest that domestication and prolonged breeding have impacted the vertically transferred bacteria, but only to a lesser extent have affected the soil-derived microbiota of bread wheat.

## Abstract (precis)

Genetic and physiological changes associated with plant domestication have been studied for many crop species. Still little is known about the impact of domestication on the plant-associated microbiota. In this study, we analyze the seed-associated and soil-derived bacterial and fungal microbiota of domesticated bread wheat and wild emmer wheat. We show a significant difference in the seed-associated, but not soil-derived, bacterial communities of the wheat species. Interestingly, we find less pronounced effects on the fungal communities. Overall, this study provides novel insight into the diversity of vertically transmitted microbiota of wheat and thereby contributes to our understanding of wheat as a “metaorganism.” Insight into the wheat microbiota is of fundamental importance for the development of improved crops.

## INTRODUCTION

Plants coexist with a large diversity of microorganisms. Most of the plant-associated microbiota is acquired from the environment, while a smaller component is vertically inherited: e.g., via the seed (1–3). Plant-microbe interactions range from pathogenic (4, 5) or neutral (6) to beneficial (7–9), whereby the microbiota can contribute to increased nutrient uptake, stress tolerance, and disease resistance. There is growing attention to plant microbiota and its putative role in the future improvement of agricultural plant production (10, 11).

Interactions and coevolution of plants with their associated pathogens and mutualists have been intensively studied (12–15). It is well known that the plant immune system plays a fundamental role in the interaction with both pathogens and mutualists (16, 17). Consequently, genes encoding immune-related proteins coevolve with microbially produced proteins to either abort or facilitate interactions (18). While coevolution of plants with pathogens and mutualists is widely recognized, much less is known about the coevolution of plants with commensal microorganisms whose functional relevance is less understood. Likewise, to what extent these commensal microorganisms coevolve with their host is unclear.

Plant-microbe coevolution may be pronounced for seed-associated microbes that coexist with their host over multiple generations. Seeds constitute a microbial niche for dispersion and transmission over multiple host generations. Knowledge on seed-associated microbes is limited compared to our understanding of microbiota associated with other plant tissues, such as leaves and roots (19, 20). This is partly due to technical challenges of handling single seeds and the extraction of sufficient amounts of microbial DNA—for example, from very small seeds of the model species Arabidopsis thaliana. A few recent studies have investigated seed-associated microbiota with culture-dependent and -independent methods for different plant species and have provided novel insight into the seed microbiomes of different plant species (20–23). The seed-associated microbiota of wheat has been investigated with culture-dependent and -independent methods and has revealed a very low diversity of seed-associated bacteria and/or fungi (24, 25). While studies of seed-associated microbiota have identified specific taxa that are vertically transmitted, we still know little about the functional relevance of these (11). Moreover, very few studies have used comparative analyses of closely related species to address evolution of the vertically plant-transmitted microbiota.

Wheat represents one of the most important staple crops in the world. Understanding the impact of artificial plant selection during domestication and genetic plant divergence on the wheat-associated microbiota is important for the development of future wheat cultivars. Bread wheat, Triticum aestivum, was domesticated in the Fertile Crescent 10,000 to 12,000 years ago, and the domestication history has been well characterized (26–28). Moreover, the underlying genetics of wheat domestication has been described in detail, including strong reduction in genetic diversity of domesticated species, polyploidization, and strong directional selection on loci encoding desired traits (28, 29). More recently, comparative genome analyses have allowed identification of domestication signatures along the *T. aestivum* genome (30). *T. aestivum* has been dispersed worldwide with wheat cultivation and constitutes a major crop on all continents (31). Wild relatives of the wheat occur naturally in natural grassland vegetations in the Near East, including tetraploid emmer wheat, Triticum dicoccoides, diploid einkorn, Triticum boeoticum, and red wild einkorn, Triticum urartu (32, 33). The well-documented domestication history and close relatedness of wild and domesticated wheat provide a unique framework for comparative analyses of plant-associated microbial communities. Moreover, they allow us to address the evolution under cultivation of associated microbial communities.

We hypothesized that domestication and plant breeding have impacted genetic traits involved in plant-microbe interactions and microbial assembly. Notably, artificial selection and the movement of crop plants over long distances will have reduced local coadaptation and coevolution of plant-microbiota associations. We hypothesize that domestication and evolution during cultivation have altered the ability of crop plants to maintain and associate with microbiota. To address the impact of cultivation on the “core” seed-associated microbiota, we have here investigated the diversity of seed-associated bacterial and fungal colonizers of three wild wheat species, *T. dicoccoides*, *T. urartu*, and *T. boeoticum*, and domesticated wheat, *T. aestivum*, including a landrace and a cultivar. We have characterized the microbial communities of individual seeds and in seedlings propagated under axenic conditions. Moreover, we have germinated and propagated seeds of *T. dicoccoides* and *T. aestivum* in controlled experiments to ask if microbial assembly differs between the wheat genotypes when grown in natural and agricultural soils. This experiment also allowed us to address to what extent the seed-associated microbiota persists in roots and leaves in the presence of soil-derived colonizers. Our findings suggest that a main consequence of plant domestication and cultivation on the seed-associated microbiota is a decreased diversity of seed-derived bacterial taxa colonizing the leaves and roots of the bread wheat species *T. aestivum* compared to the wild relative *T. dicoccoides*. The effect of host species is considerably less pronounced for fungal symbionts. This study is, to the best of our knowledge, the first study demonstrating the impact of domestication and cultivation on the diversity of vertically transmitted microbiota of wheat.

## RESULTS

### The overall diversities of seed-associated microbial communities are similar between domesticated wheat and wild wheat.

To compare the diversities of seed-associated microbiota between domesticated wheat and wild wheat, we first used the three wild wheat species *T. dicoccoides*, *T. urartu* , and *T. boeoticum* and two genotypes of the domesticated wheat *T. aestivum*—one landrace from Turkey and an inbred cultivar from Germany (see Table S1 and Fig. S1 in the supplemental material). For *T. dicoccoides*, we used genotypes from four different populations and for *T. boeoticum* two different populations in southeast Turkey (Table S1).

10.1128/mBio.02637-20.2 FIG S1 Location of the seed collections. (A) Wheat seeds were collected in Turkey and Germany. (B) Location of fields from the southeast region of Turkey and north Germany where seeds were collected. An enlarged version of the locations is depicted to make visualization easier. Download FIG S1, EPS file, 1.9 MB. Copyright © 2020 Özkurt et al. 2020 Özkurt et al. This content is distributed under the terms of the Creative Commons Attribution 4.0 International license.

10.1128/mBio.02637-20.8 TABLE S1 Detailed information about the wheat collections. Download Table S1, XLSX file, 0.04 MB. Copyright © 2020 Özkurt et al. 2020 Özkurt et al. This content is distributed under the terms of the Creative Commons Attribution 4.0 International license.

**FIG 1:**
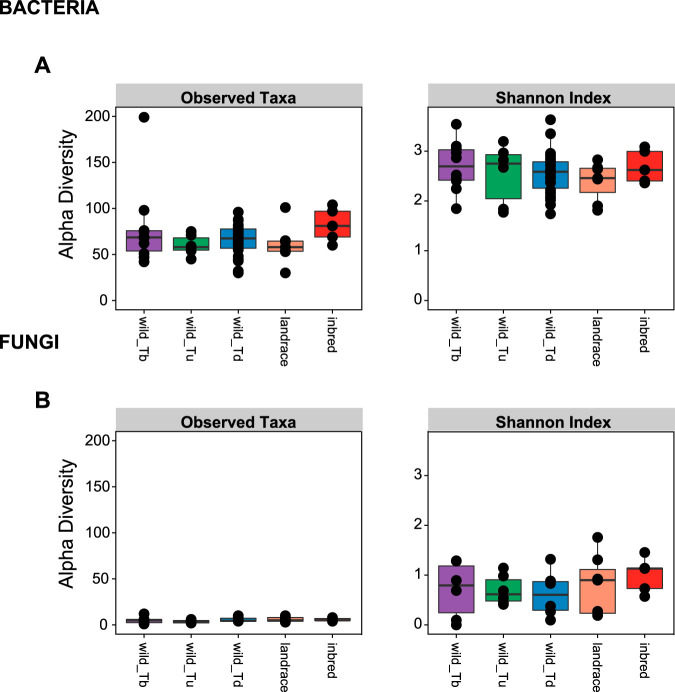
Estimates of α diversity in the seed-associated microbiota of different wheat genotypes show similar microbial feature diversities in domesticated and wild wheat. Shown is the α diversity of (A) bacterial and (B) fungal features in the seeds of different wheat species, including *T. boeoticum* (wild_Tb), *T. urartu* (wild_Tu), *T. dicoccoides* (wild_Td), and *T. aestivum* from Turkey (landrace) and Germany (inbred). Each dot in the box plots shows the microbial feature diversity of a single seed. Pairwise comparisons of α diversity showed no significant difference between wheat genotypes.

Measures of α diversity (within-sample diversity) in seeds show an overall low diversity of microbial features and notably a low diversity of seed-associated fungal features compared to other plant tissues, like leaves and roots (see below). Hereby we find across 58 individual seed samples an average richness of 68.7 bacterial and 5.3 fungal features (average Shannon index values of 2.6 and 0.8 for bacteria and fungi, respectively) (Fig. 1A and B). We observed no difference in the α diversities of microbial features associated with seeds of wild and domesticated wheat (Conover’s test, richness of *P*_bacteria_ = 0.8029 and *P*_fungi_ = 0.1924; Shannon diversity, *P*_bacteria_ = 0.6728 and *P*_fungi_ = 0.2530).

Taken together, our estimates of α diversity in different wheat genotypes demonstrate relatively low microbial diversity in individual seeds of wheat. Furthermore, we find that domestication has not entailed a loss of diversity in the microbial community associated with seeds of domesticated wheat in comparison to the communities associated with seeds of wild wheat.

### The taxonomic composition of seed microbiota differs among wheat genotypes.

Next, we investigated the composition of microbial communities associated with the wheat seeds. Comparisons of β diversity (between-sample variation) showed that the seed-associated bacterial and fungal communities do not cluster according to the wheat genotype (see Fig. S2 in the supplemental material). However, the seed-associated microbial communities of *T. aestivum* accessions from Germany and Turkey are more similar, although the first represents a highly inbred modern cultivar and the second a local Turkish landrace.

10.1128/mBio.02637-20.3 FIG S2 Bray, UniFrac, and Jaccard distance-based PCoA of the microbial community in seeds and axenic seedlings. (A) Bray-Curtis PCoA seed-associated bacteria and fungi. (B) Unweighted UniFrac PCoA of seed-associated bacteria. (C) Weighted UniFrac PCoA of seed-associated bacteria. (D) Jaccard PCoA of seed-associated bacteria and fungi. (E) Unweighted UniFrac PCoA of axenic seedling-associated bacteria. (F) Jaccard PCoA of axenic seedling-associated bacteria and fungi. (G) Weighted UniFrac PCoA of axenic seedling-associated bacteria. Download FIG S2, EPS file, 2.6 MB. Copyright © 2020 Özkurt et al. 2020 Özkurt et al. This content is distributed under the terms of the Creative Commons Attribution 4.0 International license.

**FIG 2:**
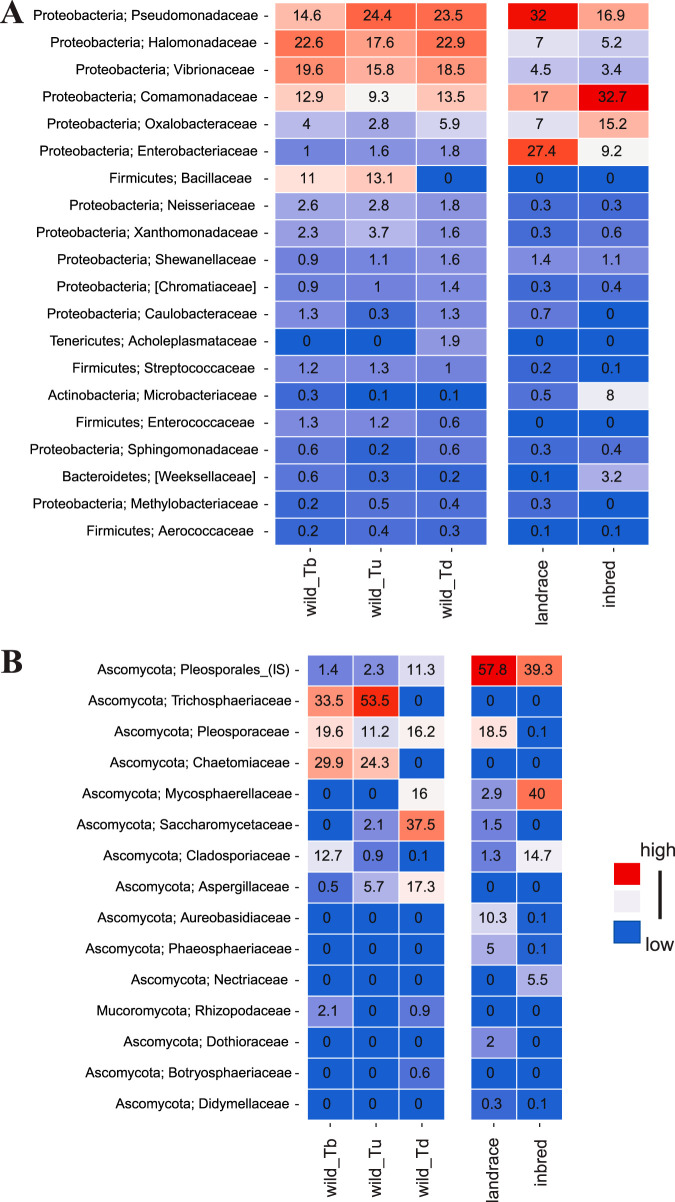
Compositions of the seed-associated microbiota across different wheat genotypes. Shown are the mean relative abundances of the (A) 20 most abundant bacterial features and (B) 15 most abundant fungal features at the family level in seeds of the different wheat genotypes of *T. boeoticum* (wild_Tb), *T. urartu* (wild_Tu), *T. dicoccoides* (wild_Td), and *T. aestivum* from Turkey (landrace) and Germany (inbred). Color for each feature ranges from blue (minimum of 0) to red with higher relative abundance values. IS, *incertae sedis* taxa.

We further characterized and compared the identities and abundances of microbial taxa. We found a considerable variability among replicate seeds of the same wheat genotype. On average, only 12.1% of bacterial features at the family level exist in all replicates of the same wheat genotype. This high between-seed variability and the sample size did not allow us to identify potential hub species in a microbial network analysis. Instead, we aggregated the assigned taxonomy of each microbial feature to the family level. First, we assessed the distribution of major bacterial groups associated with seeds of the different wheat genotypes. Hereby, the wheat seed microbiota was mostly dominated by *Proteobacteria* and to a lesser extent by *Firmicutes*, *Actinobacteria*, and *Bacteroidetes* (Fig. 2A). These results are in accordance with previous studies of seed-associated bacteria of crops (e.g., maize, barley, and rice) (34–36) and non-crop plants (e.g., radish) (2, 37). However, at lower taxonomic levels, we observed differences in abundances of several bacterial taxa among the different wheat genotypes (Fig. 2A). For example, members of the *Halomonadaceae* family, including bacteria known to promote plant salt tolerance and growth (38), represent a substantial proportion of the bacterial community in the seeds of wild wheat (17.6 to 22.9%) but only a smaller proportion of the domesticated wheat seed microbiome (5.2 to 7%).

Among the fungal taxa, we also found considerable variability among replicate seeds of the same wheat genotype. (On average, 17.4% of fungal features at the family level exist in all replicates of the same wheat genotype.) Fungal communities were dominated by Ascomycetes (Fig. 2B). Notably fungi in the order Pleosporales are abundant in the wheat seeds, including species of *Alternaria* that are highly abundant in seeds of *T. aestivum* and were previously also shown to dominate wheat endophyte communities (25). Notably, Trichosphaeriaceae and Chaetomiceae were detected to be the two most prevalent fungal families in *T. boeoticum* (33.5 and 29.9%) and *T. urartu* (53.5 and 24.3%), but were not detected in other wheat genotypes.

These findings indicate that although domestication has a minor effect on the overall microbial community richness, it may have impacted the relative abundances of the seed-associated microbiota.

### Bacterial communities that colonize axenic seedlings are more diverse in wild wheat in comparison to domesticated wheat.

A part of the seed-associated microbiota colonizes the plant seedling after seed germination (2, 24). In order to compare microbial diversities and community compositions of seedling colonizers in domesticated wheat and wild wheat, we set up an experiment using seeds of the German *T. aestivum* inbred cultivar, the Turkish *T. aestivum* landrace, and the Turkish *T. dicoccoides* genotypes. We germinated surface-sterilized seeds and propagated them under sterile conditions. Two weeks after seed germination, we harvested leaves and roots of the seedlings. Finally, we processed a total of 32 plant samples consisting of roots and leaves (4 to 8 replicates for each plant tissue of each wheat genotype) and used these samples to profile bacterial and fungal communities.

**FIG 3:**
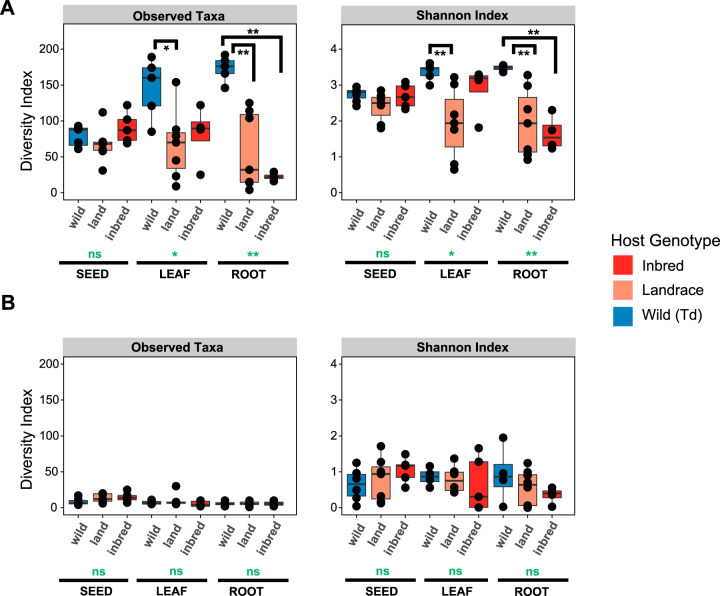
Significantly more diverse bacterial but not fungal communities are colonizing the wild wheat. Diversity of microbial features in different tissues of axenically grown wheat. Shown is the α diversity of (A) bacterial and (B) fungal taxa in seeds, leaves, and roots of the wild wheat *T. dicoccoides* (wild) and *T. aestivum* from Turkey (landrace) and Germany (inbred), respectively. Each dot in the box plots shows the microbial feature diversity of a single replicate. Global *P* values for each tissue based on Kruskal-Wallis test are shown in green, and *P* values of pairwise comparisons based on Conover’s test are in black. *, *P* < 0.05; **, *P* < 0.005; ns, nonsignificant.

Analyses of the bacterial feature diversity revealed a total of 589 and 632 different bacterial features in leaves and roots (after filtering and rarefaction) (see Table S2 in the supplemental material). The analysis revealed that bacterial communities associated with the roots of *T. dicoccoides* (“wild_Td”) are significantly more diverse than the communities associated with the Turkish and German *T. aestivum* genotypes (landrace and inbred, respectively) (pairwise α diversity comparisons, Conover’s test, richness of *P*_wild_Td+landrace_ = 0.0023 and *P*_wild_Td+inbred_ = 0.0023; Shannon index, *P*_wild_Td+landrace_ = 0.0021 and *P*_wild_Td+inbred_ = 0.0027). Additionally, the leaves of *T. dicoccoides* hosted more diverse bacterial communities compared to domesticated wheat from Turkey (Conover’s test, richness, *P* = 0.0280; Shannon index, *P* = 0.0066) (Fig. 3A). Taken together, the diversity of seed-associated bacterial features colonizing the leaves and roots is significantly higher in wild wheat compared to the two genotypes of domesticated wheat.

10.1128/mBio.02637-20.9 TABLE S2 Summary of the sampling and sequencing data. Download Table S2, XLSX file, 0.04 MB. Copyright © 2020 Özkurt et al. 2020 Özkurt et al. This content is distributed under the terms of the Creative Commons Attribution 4.0 International license.

We obtained in total only 98 and 74 unique fungal features in leaves and roots, respectively (after filtering and rarefaction). Our results revealed no significant differences in the feature diversity of fungal colonizers between domesticated and wild wheat, suggesting that different processes determine the colonization of bacterial and fungal endophytes transmitted by seeds (Fig. 3B).

**FIG 4:**
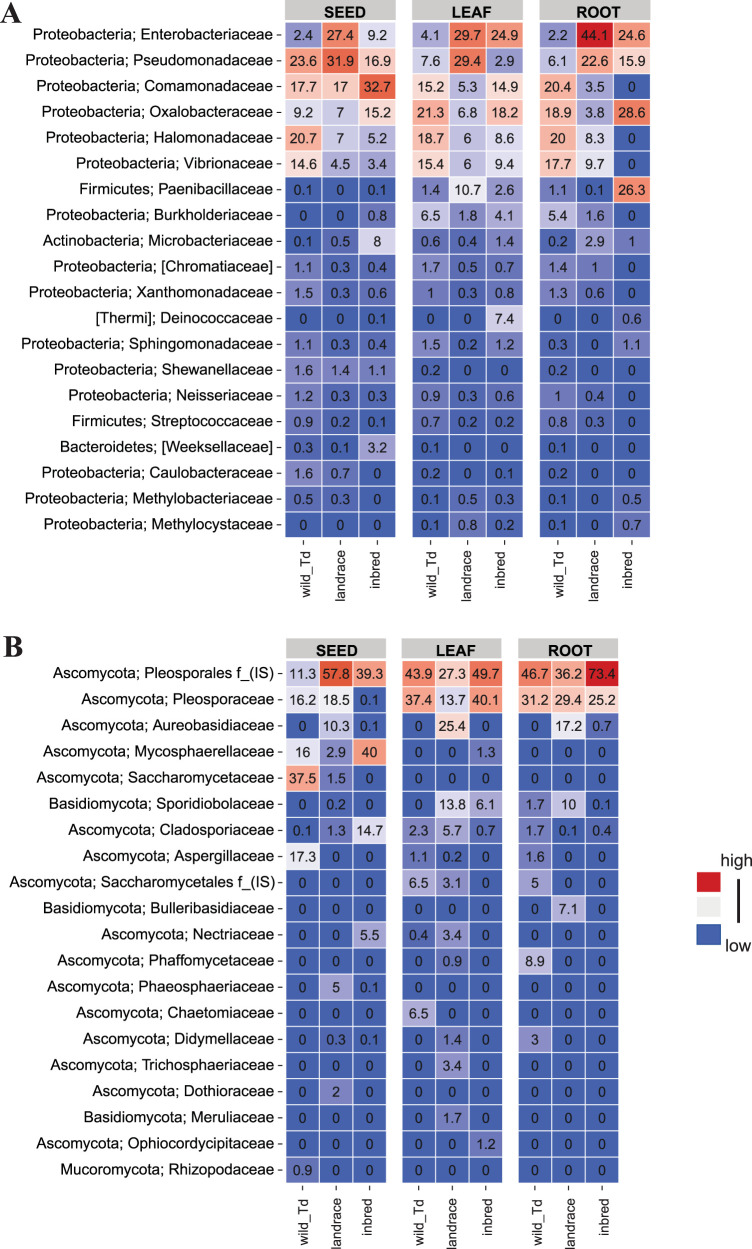
Axenic seedlings of wheat are colonized by diverse bacterial and fungal taxa. Shown are the mean relative abundances of the 20 most abundant (A) bacterial and (B) fungal features at the family level in seeds, leaves, and roots of the German *T. aestivum* genotype (inbred), the Turkish *T. aestivum* genotype (landrace), and the wild wheat *T. dicoccoides* genotype (wild_Td), respectively. Colors for each taxon illustrate relative abundance and range from blue (minimum of 0) to red with higher relative abundance values. IS, *incertae sedis* taxa.

We next compared the identities and abundances of the microbial communities of seeds and seedlings. Overall, the same bacterial and fungal phyla were dominant in seeds as well as in leaves and roots; however, we observed several significant shifts in microbial abundance (Fig. 4A). For example, *Comamonadaceae*, *Halomonadaceaea*, *Vibrionaceae*, and several other bacterial families enriched in seeds of both wild and domesticated wheat did not colonize roots of the German *T. aestivum* accession. Furthermore, *Paenibacillaceae* were only present at very low abundance (0 to 0.1%) in seeds, but were found to be a dominant colonizer of roots of *T. aestivum* from Germany (26.3%).

Fungal Ascomycota taxa were the most abundant in the wheat seedlings (Fig. 4B). Notably, Pleosporales were abundant colonizers of seedlings of both wild and domesticated wheat from Turkey and Germany. Aureobasidiaceae were abundant only in the seedlings of *T. aestivum* from Turkey (25.4% in leaves and 17.2% in roots), but not in the two other wheat genotypes. Other abundant seed-associated fungi were not found to colonize the leaves and roots of the wheat seedlings. For example, Mycosphaerellaceae (40% in *T. aestivum* from Germany), Saccharomycetaceae (37.5% in *T. dicoccoides*), and Aspergillaceae (17.3% in *T. dicoccoides*), found to be abundant in the seed-associated communities, were either absent or only present at low relative abundance in the seedlings. Together, these results demonstrate a difference in the assembly of seed-associated bacterial, but not fungal communities in wild and domesticated wheat seedlings. Moreover, our results indicate that more diverse seed-transmitted microbial communities are sustained in the roots and leaves of wild wheat seedlings compared to domesticated wheat seedlings.

**FIG 5:**
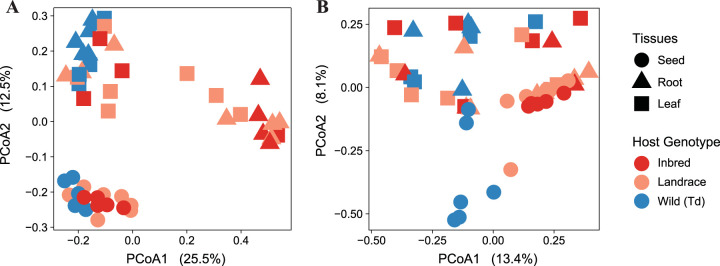
More similarity of bacterial community composition between replicates of the wild wheat seedlings compared to the domesticated wheat seedlings. Shown are Bray-Curtis distance metrics-based PCoA of (A) bacterial and (B) fungal communities of each seed, leaf, and root sample from the axenic experiment.

We further examined between-sample variation by computing Bray-Curtis and Jaccard and unweighted UniFrac distance metrics (Fig. 5; Fig. S2). Our results show that replicates of seed-associated bacterial communities of the wild wheat and domesticated wheat from Turkey and Germany cluster together in the principal-coordinate analyses (PCoA) (Fig. 5A; Fig. S2D and E). However, seed-derived bacterial colonizers in the seedlings of *T. dicoccoides* are distinct from the seed-associated community (Fig. 5A), and there is less variation among replicates of root and leaf communities. In contrast, in the domesticated *T. aestivum* wheat from Germany and Turkey, we observed more heterogeneous microbial communities associated with the leaves and roots (Fig. 5A; see Fig. S3 and S4A in the supplemental material) than in the wild wheat. Additionally, we compared variabilities in fungal communities among seed and seedling replicates. Different than the bacterial communities, we found more variability among replicates of seed-associated fungi as well as colonizers of roots and leaves for both domesticated and wild wheat genotypes (Fig. 5B; Fig. S2D and S4B). Overall, these findings support that different processes govern the assembly of bacterial and fungal communities in seeds and seedlings of wheat.

10.1128/mBio.02637-20.4 FIG S3 PCoA plots based on β diversity distances of bacterial communities in the axenic leaves and roots of wild and domesticated wheat. (A) Bray-Curtis, (B) Jaccard, (C) weighted UniFrac, and (D) unweighted UniFrac distance-based PCoA of bacterial communities in axenic seedlings. Download FIG S3, EPS file, 1.1 MB. Copyright © 2020 Özkurt et al. 2020 Özkurt et al. This content is distributed under the terms of the Creative Commons Attribution 4.0 International license.

10.1128/mBio.02637-20.5 FIG S4 Estimated pairwise Bray-Curtis distances of microbial communities in the axenic leaves and roots of wild and domesticated wheat. (A) Bacterial and (B) fungal communities of replicate seedlings of each wheat genotype and for each tissue. Global *P* values for each tissue based on Kruskal-Wallis test are shown in green, and *P* values of pairwise comparisons based on Conover’s test are in black. **, *P* < 0.005; ***, *P* < 0.0005; ns, nonsignificant. Download FIG S4, EPS file, 1.9 MB. Copyright © 2020 Özkurt et al. 2020 Özkurt et al. This content is distributed under the terms of the Creative Commons Attribution 4.0 International license.

### The assembly of the soil-derived root microbiota is independent of the wheat genotype.

In their natural environment, plant seedlings are also colonized by microorganisms from the soil (10, 39). To investigate if seedlings of *T. dicoccoides* and *T. aestivum* assemble different microbial communities from soil, we set up an experiment with the same wheat genotypes as used above. We grew seedlings of the three wheat genotypes in two different soils: an agricultural soil from Germany and a natural soil obtained from a location in the southeast region of Turkey close to the sampling site where the wild wheat accessions were obtained. This experimental setup allowed us not only to assess differences between wheat genotypes, but also to address the relevance of soil type in microbial assembly of wild and domesticated wheat genotypes. To this end, the agricultural soil represents a “foreign” soil in combination with the wild wheat seeds, and the natural Turkish soil represents a “native soil.” We propagated the three wheat genotypes (*T. dicoccoides* from Turkey and *T. aestivum* from Turkey and from Germany) independently in the agricultural and natural soils (6 to 8 replicate plants per wheat-soil combination). The seeds used here were surface sterilized as in the experiments described above.

**FIG 6:**
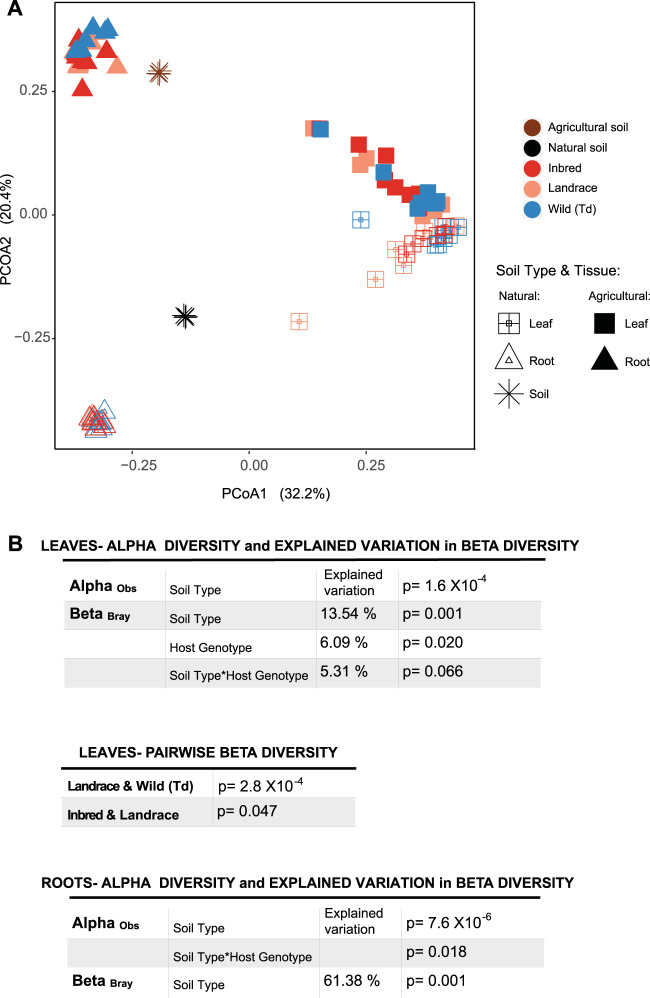

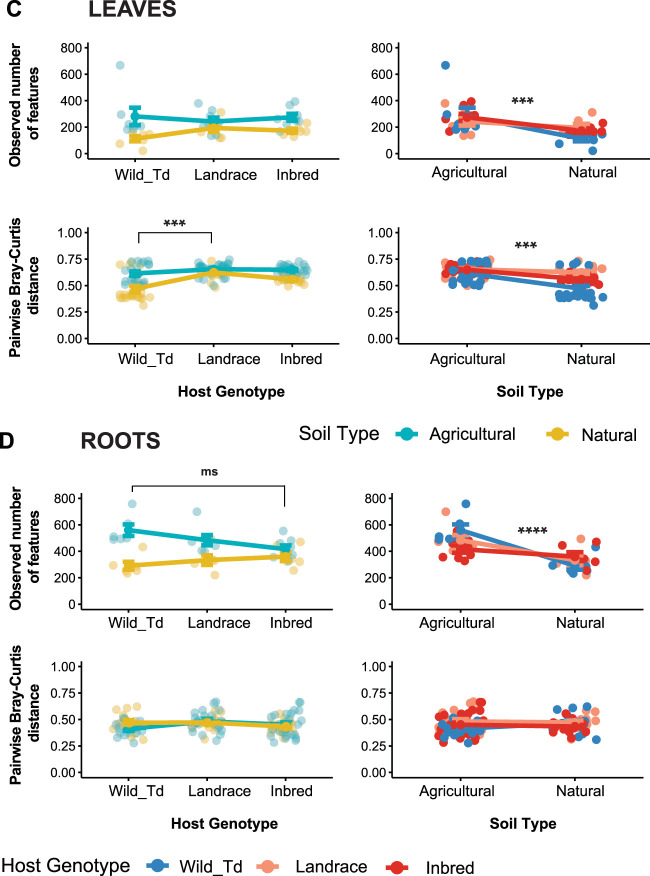
Soil type is a main determinant of microbial diversity in wheat seedlings. (A) Bray-Curtis distance-based PCoA of bacterial communities of plants grown in soil. (B) Summary statistics for the β diversity (PERMANOVA to estimate the explained variation by each factor and their interactions) and α diversity comparisons of the bacterial communities (two-way ANOVA as the global test and Tukey’s honest test as the *post hoc* test). Only significant *P* values are indicated. (C and D) Interaction plots showing α and β diversity comparisons of bacterial communities in (C) leaves and (D) roots of different wheat genotype grown in different soil types. Each dot in the α diversity comparisons shows the microbial feature diversity of a single replicate. In the box plots of β diversity comparisons, each dot shows a single pairwise comparison between two replicate plants. Tukey’s honest test after two-way ANOVA was performed for pairwise diversity comparisons. ms, marginally significant.

Remarkably, we found no prominent difference among wild and domesticated wheat species in terms of microbiota assembly. First of all, our results reveal that soil type rather than plant genotype is a main determinant of bacterial community structure in roots of wheat seedlings (using a permutational analysis of variance [PERMANOVA], explained by 61.38% of the between-sample variation; *P* = 0.001) (Fig. 6A and B). Also, soil type is the main factor of the bacterial α diversity in both leaves and roots (*P*_ANOVA_ = 1.6 × 10^−4^ and 7.6 × 10^−6^ and *P*_Shapiro_ = 0.093 and 0.052, respectively) (Fig. 6B). However, for the leaf-associated microbial communities, we found that also the wheat genotype explains a significant proportion of the between-sample variation (for soil type, 13.54%, *P* = 0.001; for wheat genotype, 6.09%, *P* = 0.020; and for the interaction of soil and wheat genotypes, 5.31%, *P* = 0.066) (Fig. 6).

We also collected microbiota data from pure soil samples, and found that the two different soils were similar with respect to the diversity of bacterial features (natural soil, richness = 789.6, Shannon index = 6.16; agricultural soil, richness = 776.0, Shannon index = 6.24). Nevertheless, leaves and roots of plants grown in the agricultural soil were, in general, colonized by more diverse bacterial communities (Fig. 6C and D). In contrast to the axenic experiment, we did not detect a difference in α diversities between leaves of wild and domesticated wheat species when seedlings were propagated in the soil. However, in accordance with our observations from the axenic experiment, we observed less variation between replicates of *T. dicoccoides* compared to the landrace of *T. aestivum* from Turkey (*P* = 2.8 × 10^−4^) in leaves (Fig. 6B). On the other hand, we observed a significant difference in α diversities between root-associated microbial communities of *T. dicoccoides* and the German accession of *T. aestivum* propagated in the agricultural soil (*P* = 0.055) (Fig. 6D). Hereby, the diversity of bacterial features is significantly higher in roots of wild wheat compared to domesticated wheat.

The composition of fungal communities was assessed only in roots of the three wheat genotypes (see Fig. S5 in the supplemental material). In line with previous literature, our results show that soil type rather than the wheat genotype is the main determinant of variation in fungal communities associated with plant roots (PERMANOVA, 8.52% of the between-sample variation; *P* = 0.001) (Fig. S5A and B). Moreover, in contrast to the bacteria, the wild wheat was colonized by less homogenous fungal communities compared to the domesticated wheat in both soil types (Fig. S5B). Also, based on analyses of pairwise Bray-Curtis distances, we found that fungal communities are more similar among replicates when seedlings were propagated in the natural soil compared to the agricultural soil (Fig. S5C). However, we did not observe a significant effect of soil type on either wheat genotype in fungal α diversity. Taken together, soil type but not wheat genotype had a strong impact on the differences in microbial β diversity and community composition of fungal wheat colonizers.

10.1128/mBio.02637-20.6 FIG S5 Bray-Curtis distance-based PCoA interaction plots and compositions of the fungal communities of roots grown in the soil from agriculture and wild. (A) Data based on the Bray-Curtis distances of root-associated fungal community of the replicate plants grown in two different soil types. (B) Summary statistics for the β diversity (PERMANOVA to estimate the explained variation by each factor and their interactions) and α diversity comparisons of the bacterial communities (two-way ANOVA as the global test and Tukey’s honest test as the *post hoc* test). Only significant *P* values are indicated. (C) Interaction plots show α and pairwise β diversity comparisons (i.e., pairwise Bray-Curtis distances) of fungal communities in roots of different wheat species grown in agricultural and natural soils: *T. dicoccoides* (wild_Td) and *T. aestivum* from Turkey (landrace) and Germany (inbred). (D) Mean relative abundances of the most abundant 20 fungal features at the family level in roots of different wheat genotypes grown in agricultural and natural soils. Color for each feature ranges from blue (minimum of 0) to red with higher relative abundance values. IS, *incertae sedis* taxa. Download FIG S5, PDF file, 0.6 MB. Copyright © 2020 Özkurt et al. 2020 Özkurt et al. This content is distributed under the terms of the Creative Commons Attribution 4.0 International license.

**FIG 7:**
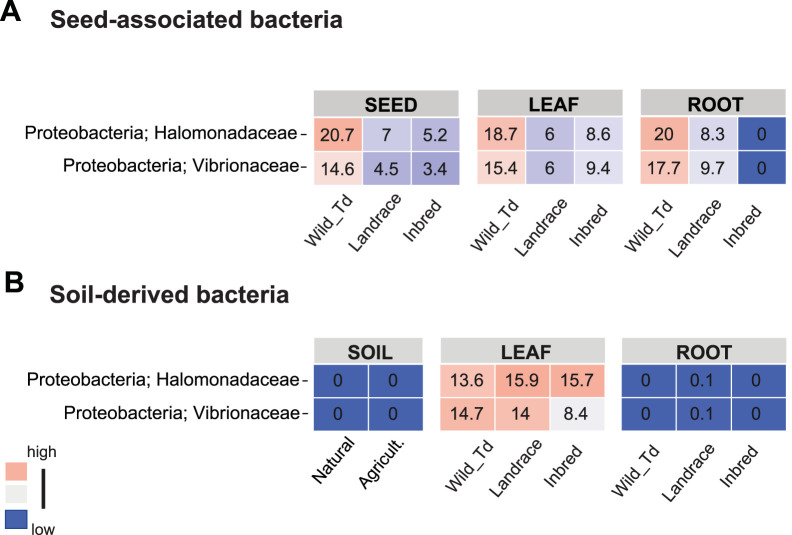
*Halomonadaceae* and *Vibrionaceae* are potential seed-derived microbes persisting in the leaves of soil-grown seedlings. (A) Mean relative abundances of *Halomonadaceae* and *Vibrionaceae* in the seeds and axenic seedlings (B) Mean relative abundances of *Halomonadaceae* and *Vibrionaceae* in the natural and agricultural soils and in the seedlings grown in these soils.

We next compared the identities and abundances of microbial taxa in the seedlings of wheat propagated in soil. Clearly, roots and leaves exhibited distinct bacterial compositions compared to the bacterial communities of the bulk soil, implying specificity related to plant colonization (Fig. 6A; see Fig. S6A in the supplemental material). However, in general microbial compositions were similar among the three different wheat genotypes when grown in the same soil type (Fig. S6). Basically, roots were dominated by two bacterial families, *Oxalobacteraceae* (18.6 to 26.5%) and *Streptomycetaceae* (27 to 32.6%) (Fig. S6A), where *Streptomycetaceae* were the dominant colonizer of roots when seedlings were growing in the agricultural soil (40.2 to 49.7%) (Fig. S6C). On the other hand, leaves were colonized by other bacterial families: *Oxalobacteraceae* (12.2 to 22.2%), *Comamonadaceae* (7.4 to 19.9%), *Rhizobiaceae* (12.1 to 21.3%), *Halomonadaceae* (13.6 to 15.9%), and *Vibrionaceae* (8.4 to 14.7%). Notably, the two bacterial families *Halomonadaceae* and *Vibrionaceae* were highly abundant phyllosphere colonizers in the leaves of seedlings grown in both soil types, although they were not detected in the bulk soil (Fig. 7B). On the other hand, these bacteria were also prevalent members of the axenic phyllosphere bacterial community, suggesting that these bacterial taxa may have originated from the seeds (Fig. 7A) and persist in the leaves after soil microbiome colonization (Fig. 7B).

10.1128/mBio.02637-20.7 FIG S6 Compositions of the bacterial communities in seedlings colonized by the soil-derived microbiota. Shown are the mean relative abundances of the 20 most abundant bacterial features at the family level. (A) Bacterial communities in different tissues. (B) Leaf-associated bacterial communities in plants grown in agricultural and natural soils. (C) Root-associated bacterial communities in plants grown in agricultural and natural soils. Results are shown for *T. dicoccoides* (wild_Td) and *T. aestivum* from Turkey (landrace) and Germany (inbred). Download FIG S6, PDF file, 0.4 MB. Copyright © 2020 Özkurt et al. 2020 Özkurt et al. This content is distributed under the terms of the Creative Commons Attribution 4.0 International license.

Also, the fungal communities were similar among the different wheat genotypes when grown in the same soil type (Fig. S5D). Fungi of the most prevalent member of seed-associated fungal communities, Pleosporaceae, were still detectable in the roots of plants propagated in the natural soil (10.8 to 16.6%). However, plants grown in the agricultural soil were mostly colonized by Pseudeurotiaceae (28.4 to 33.9%).

## DISCUSSION

Plant domestication has entailed a significant loss of genetic diversity, as well as physiological and anatomical changes in crop species (e.g., see reference 18). In this study, we have addressed how domestication of wheat, involving strong selection and polyploidization, has affected the seed-associated microbiota composition and the assembly of environmental (acquired from the soil) microbial communities. We combined experimental assays with microbiota profiling of a collection of modern bread wheat and wild wheat genotypes from a region in the Fertile Crescent. To describe microbial diversity for individual seeds, we optimized our protocols of DNA extraction and microbial amplification, allowing us to obtain a high-resolution profile of diversity of bacterial and fungal features in individual wheat seeds (see Text S1 and Table S2 in the supplemental material).

10.1128/mBio.02637-20.1 TEXT S1 Supplemental methods. Download Text S1, DOCX file, 0.03 MB. Copyright © 2020 Özkurt et al. 2020 Özkurt et al. This content is distributed under the terms of the Creative Commons Attribution 4.0 International license.

We show that wheat domestication, plant polyploidization, and evolution during cultivation did not entail a modification of the overall seed-associated microbial diversity of wheat. However, we show a significant difference in the feature diversities and compositions of seed-derived microbial colonizers of wheat seedlings. An underlying assumption in our study of the seed-associated microbiota is that a significant proportion of these microorganisms later represent endophytes in the wheat plant. To qualitatively and quantitatively characterize the early seedling colonizers, we propagated seedlings under sterile conditions and assessed microbial diversity in leaves and roots of the young seedlings. Interestingly, we find a higher similarity of microbial communities between seeds of *T. aestivum* from Turkey and Germany than between seeds of *T. aestivum* and *T. dicoccoides* from the region spanning Fertile Crescent in Turkey. This indicates a genetic determinant of the microbiota assembly. Another striking finding from this experiment was a higher diversity of bacterial features colonizing the wild wheat *T. dicoccoides* compared to *T. aestivum*. This difference may reflect that a larger diversity of microorganisms in seeds of the wild wheat species is adapted to an endophytic lifestyle. However, we note that the composition of seed-associated microbiota may have been partly determined by the environment of the mother plant from seeds collected in the field. Therefore, in future studies these findings should be validated in common garden experiments where the environment of each plant generation is controlled and equal for each wheat genotype. Also, we note that our analyses only detect changes in the relative abundance of microbial taxa, and we may therefore fail to detect some shifts in taxonomic differentiation.

In contrast to the bacterial communities, we did not detect a difference in fungal communities of wild and domesticated wheat seedlings. Other studies have assessed diversity of fungal endophytes and also shown relatively low taxonomic diversity (1, 39, 40) and little difference between domesticated and wild species (1). We note that the fungal diversity assessed in our study in wheat seeds overall is very low and therefore reduces the statistical power to detect enriched and deprived taxa.

It was recently shown that perturbations of the host (e.g., disease) change the associated microbiota toward a more stochastic composition in animals (41–43), a phenomenon that is termed the “Anna Karenina principle” (44). The Anna Karenina principle claims that stressors decrease the ability of the host to regulate its microbial composition as a result of compromised host immunity (44). For example, autoimmune dysregulation in patients with type1 diabetes increases the stochasticity of bacterial community composition (45). Here, we speculate that domestication likewise may have entailed less selective constraints on plant traits that contribute to microbial assembly. Consequently, in line with the Anna Karenina principle in animal microbiomes, nondeterministic events could play a larger role in the assembly of microbial communities associated with domesticated plants.

A growing body of evidence suggests that different layers of the plant immune system play significant roles in shaping the plant microbiota (46). Plant receptors that are involved in microbial recognition and “management” involve pathogen recognition receptors (PRRs) and nucleotide-binding leucine-rich repeat receptor (NB-LRR) proteins (47). Wheat domestication and polyploidization may have conferred a change in the composition and diversity of these immune-related proteins and thereby indirectly impacted the assembly of the wheat microbiome. Comparative genome studies of domesticated and wild wheat have identified signatures of domestication in the *T. aestivum* genome, which may correlate with microbial community assembly, including changes in the repertoire of genes involved in immune signaling, hormone production, and metal accumulation (48). We speculate that different microbial community compositions in German and Turkish *T. aestivum* variants and the wild relative *T. dicoccoides* reflect genetic differences related to the plant immune system. We note that such changes notably have impacted the compositions of bacterial communities and to a much lesser extent those of fungal communities. Interestingly, we observe no significant differences in α diversity among seed- and soil-derived fungal communities of the wild and domesticated wheat genotypes. However, different from the bacterial community, the soil-derived fungal community is less homogenous in wild emmer wheat compared to the landrace and inbred bread wheat. This suggests that different traits and mechanisms are responsible for the assembly of bacterial and fungal plant-associated communities. However, the amplicon-based analyses applied here do not provide sufficient resolution to confirm this hypothesis. The low percentage of microbial features shared among replicates of the plant tissues prevented us from performing network analyses of co-occurrence. Thus, more detailed analyses of microbial diversity (e.g., based on metagenome sequencing or microbial population genomic data) are needed to study plant-microbe coadaptation and microbe-microbe interactions.

Domestication and plant breeding have involved strong artificial selection of desired crop traits. For several domesticated species, it has been demonstrated that a negative consequence of domestication is a severe loss of genetic variation and an accumulation of deleterious mutations (29, 49, 50). These “domestication costs” may have reduced local adaptation of crop plants to their environment, including the local environmental microbiota. Our microbial profiling of leaves and roots of the wheat seedlings shows that soil rather than plant genotype determines the composition of root-associated bacterial communities. These findings suggest that the “plant-selected” proportion of the soil-derived microbiota overall is small. Interestingly, we observe a stronger effect of the plant genotype on the bacterial phyllosphere community than on the root-associated bacterial communities. Furthermore, we detect two bacterial families, *Halomonadaceae* and *Vibrionaceae*, as being abundant in the wheat seeds and as well as prevalent colonizers of the phyllosphere. We do not detect these bacterial families in the agricultural and natural soils and therefore consider the seeds as the only possible input for the seedling-associated microbiome. This observation indicates that vertically transmitted microbial taxa can be important components of plant microbial communities.

In conclusion, the present study provides new insights into the microbial community composition and colonization of domesticated and wild wheat. Our findings indicate different dynamics in the assembly of fungi and bacteria in wheat seedlings and also suggest an effect of plant domestication. We speculate that different patterns of microbiota assembly reflect variation in immune-related pathways that contribute to microbiota assembly (46). Future studies should identify the underlying genetic basis of microbiota assembly in wheat as well as the specific relevance of the seed-associated microbiota. Future crop breeding strategies should account for microbial diversity and the ability of crops to assemble and maintain beneficial microbial communities. Such efforts will rely on research of plant-microbe coadaptation and the underlying mechanisms that determine microbial plant colonization.

## MATERIALS AND METHODS

### Experimental design.

First, we investigated bacterial and fungal communities associated with individual seeds of wild and domesticated wheat. To this end, we processed individual seeds from the wild wheat species *T. dicoccoides*, *T. urartu*, and *T. boeoticum* and from the domesticated wheat *T. aestivum*, including a Turkish landrace and an inbred cultivar from plant breeders in Germany.

Next, we characterized the seed-derived bacterial and fungal communities associated with leaves and roots of axenically grown seedlings. To this end, we germinated surface-sterilized seeds of the wild wheat *T. dicoccoides* and the domesticated wheat *T. aestivum*, including the landrace and inbred cultivars, under axenic conditions.

Lastly, we addressed the “soil-derived” component of the wheat seedling microbiota. Therefore, we germinated surface-sterilized wheat seeds of *T. dicoccoides* and *T. aestivum* in two different soil types. Details of the experimental protocols and approaches are explained in the following sections.

### Seed collections.

Our study built on a unique collection of wheat material, including the three wild species *T. dicoccoides* (2*n* = 28), *T. boeoticum* (2*n* = 14), and *T. urartu* (2*n* = 14) collected in the Near East and the domesticated bread wheat *T. aestivum* (2*n* = 42) collected in the Near East and North Germany (Fig. S1 and Table S1). We here refer collectively to these wheat species and cultivars as wheat “genotypes.” More precisely, the wild wheat species were sampled in a region in southeast Turkey, a region located in the Fertile Crescent known to be the natural environment of these three wild wheat progenitors. Moreover, the region is considered to be a site of early domestication and cultivation of the bread wheat *T. aestivum* (33). Our seed collection of the wild wheat represents two geographical populations of central-eastern Turkish-Iraqi wheat (32). Seeds of the wild wheat were collected from one of the centers of massive stands in Karacadağ (provinces of Şanlıurfa and Diyarbakır) and Kartal-Karadağ (province of Gaziantep) in the southeast region of Turkey at different nearby fields over 3 years: 2004, 2005, and 2006 (Table S1 and Fig. S1) (32).

Seeds of the domesticated wheat *T. aestivum* were obtained from a local farm located in a region spanning Fertile Crescent in Turkey where the wild wheat was collected. The *T. aestivum* genotype from Turkey is a winter wheat and local landrace of Kışlak, a province of Hatay. During cultivation, this wheat is not treated with chemicals by the farmer and is treated with only with a minimum amount of fertilizer (N. Gündüz, personal communication). Also, we collected seeds from a modern winter wheat cultivar, Benchmark (IG Pflanzenzucht, Ismaning, Germany), originating from an experimental farm in Schleswig-Holstein, Germany. In contrast to the Turkish *T. aestivum*, this inbred cultivar was treated with chemicals during seed production. Seeds of both *T. aestivum* genotypes were collected in 2017.

Seeds were stored at 4°C in paper bags until further usage to prevent any effect of humidity. The comparable diversities of microbes in the seeds of wild collections from different years and compared to seeds of the German cultivar confirm a minimal impact of seed maintenance on the overall diversity that we observe (Fig. 1).

### Processing of the seeds, leaves, and roots.

To ensure isolation of only microbial DNA from the interior of seeds and tightly attached to the surface, all seeds were mildly surface sterilized before DNA extraction. Chaff was removed by hand from all the seeds before the sterilization. Seeds were surface sterilized by briefly soaking them in 0.1% Triton X, 80% ethanol (EtOH), and 1.2% bleach followed by three washes with nuclease-free water. Three randomly selected samples from the last washing were amplified and processed for sequencing. We used these samples as negative controls to ensure the efficacy of the surface sterilization.

Sterilized seeds were frozen using a Cryolys cooling unit and homogenized with a Precellys Evolution tissue homogenizer (Bertin Instruments, Montigny-le-Bretonneux, France). DNA was extracted from single seeds that originated from the wild wheat species *T. dicoccoides* (*n* = 28), *T. urartu* (*n* = 6), and *T. boeoticum* (*n* = 10) and from the domesticated wheat *T. aestivum*, including the Turkish landrace (*n* = 5) and the inbred cultivar (*n* = 7) following a phenol-chloroform extraction protocol (described in Text S1). This method was developed from a previously established protocol for *A. thaliana* (51) and here was optimized to increase the efficiency of extraction of bacterial and fungal DNA from single seeds. Three randomly selected negative controls (i.e., blanks) of DNA extraction were also processed for sequencing. Further procedures for DNA processing and sequencing are described below.

We further addressed the colonization dynamics of the seed-associated microorganisms in an *in vitro* experiment in which we germinated seeds under sterilized conditions in closed sterile jars to assess microbial diversity in leaves and roots. In brief, seeds from three wheat genotypes (*T. aestivum* from Turkey and Germany and *T. dicoccoides* from Turkey) were surface sterilized and germinated under sterile conditions with 16-h light/8-h dark cycles at 15°C (*n* = 4 to 8 per population) in a climate chamber (Percival plant growth chambers; CLF PlantClimatics GmbH, Wertingen, Germany). In sterile jars, plants were grown in a nutrient-rich PNM medium (Text S1). A sample from the medium was processed for microbial DNA amplification and was used to validate the sterility of the medium as no amplification was obtained (data not shown). Seedlings were allowed to develop for 2 weeks until the emergence of the second leaf. Although growth performance was not assessed systematically, we note that all wheat seedlings performed well in the axenic experiment, and they appeared as healthy as the plants grown in soil experiments (described below). About 6 cm of two leaves and multiple roots of 2-week-old seedlings were harvested with sterile forceps and processed for DNA extraction. DNA extraction was performed using the PowerPlant Pro DNA isolation kit (MoBio Laboratories, Heidelberg, Germany) according to the manufacturer's instructions.

### Soil experiments with wheat plants.

To address if domestication has entailed a change in the ability of plants to associate with microbial communities, we reciprocally transplanted domesticated and wild wheat (*T. aestivum* from Germany and Turkey and *T. dicoccoides* from Turkey) in a German agricultural soil (the Hohenschulen experimental farm of Christian-Albrechts University of Kiel, Germany) and a natural soil from a region of the Fertile Crescent in Turkey (Mazıdağı, Mardin). Soil samples were collected from surface layers of the field and were kept in plastic bags at 4°C until further use. Both soil types were mixed with 5% peat and sifted with a sieve. We propagated seedlings from surface-sterilized seeds in the two soil types in the climate chamber. We harvested leaves and roots as described above for the axenically propagated seedlings (*n* = 6 to 8 per combination of wheat-soil type). Additionally, three pots per soil type were filled with soil without plants and further processed as controls. The position of each pot was changed during the experiment to randomize any spatial effect. After 2 weeks, leaves and roots were harvested with sterile forceps and scissors and mildly washed with water, 1% phosphate-buffered saline (PBS) and 1% PBS plus 0.02% Tween 20 to remove loosely attached microbes and soil particles from the roots. Finally, samples were processed for DNA extraction. DNA extraction was performed using the PowerPlant Pro DNA isolation kit (MoBio Laboratories, Heidelberg, Germany) according to the manufacturer's instructions.

### Sequencing of amplicons.

The V5 to -7 sequence of the bacterial 16S rRNA (16S rRNA gene) and a sequence of the fungal ribosomal internal transcribed spacer (ITS1) region were amplified using the primer combinations B799F-B1192R and ITS1F-ITS2, respectively (52, 53). Bacterial and fungal sequences were amplified with a two-step PCR protocol. In the first step, interfering primers were utilized to enrich amplification of the 16S rRNA and prevent unintended coamplification of the host DNA. These interfering primers were originally developed for microbial community analyses of *A. thaliana* (51). Here, we modified the interfering primers to target the corresponding wheat loci and changed the PCR protocol to optimize primer interference (Text S1). In the second step of PCR, reverse primers barcoded with 12-bp indexes and unique to each sample were used as barcodes to multiplex different samples in one sequencing run (Metabion International AG, Planegg, Germany). The primer setup used here was applied from Agler et al. (26). Sequences of all the primers can be found in Table S3. Three PCR replicates for each sample were defined as technical replicates for each PCR step and subsequently merged at the end of each PCR.

10.1128/mBio.02637-20.10 TABLE S3 Sequences of the V5 to -7 and ITS1 primers used for amplification and sequencing. Download Table S3, XLSX file, 0.01 MB. Copyright © 2020 Özkurt et al. 2020 Özkurt et al. This content is distributed under the terms of the Creative Commons Attribution 4.0 International license.

Finally, DNA in amplicon libraries was quantified using the software of a Bio-Rad gel visualizer (Bio Rad, Image Lab software 5.2.1) and the Invitrogen Qubit 3.0 fluorometer (Thermo Fisher Scientific, Darmstadt, Germany). 16S and ITS amplicons were combined in equimolar concentrations in combined libraries. During DNA extraction as well as during library preparation, samples were randomized to prevent any possible batch effect. The combined libraries were paired-end sequenced for 2× 300 cycles on an Illumina MiSeq machine (Illumina, San Diego, CA, USA) at the sequencing facility of the Max Planck Institute for Evolutionary Biology, Plön, Germany.

### Data analysis.

Raw reads were demultiplexed and converted into fastq files for downstream analysis using the bcl2fastq Conversion software v2.20.0.422 of Illumina. We followed the QIIME2 version 2019.1 pipeline to preprocess and filter the fastq files (54).

For the bacterial data analysis, the DADA2 software package integrated into QIIME2 was used to correct and to truncate sequences and filter chimeric reads for 16S reads (55). For the taxonomic classification of 16S data sets, we used the Greengenes 13.8 database (56). We utilized the q2-feature-classifier plugin of QIIME2 to extract the reference sequences from the databases and train the Naïve Bayes classifier (57). We extracted the target sequence of the B799F-B1192R primer pairs from the Greengenes 13.8 database. Next, we trained the Naïve Bayes classifier based on the reference sequences and taxonomy. Finally, the resulting feature table was used to determine taxonomic relative abundances and for the subsequent statistical analyses of diversity.

For the fungal data analysis, the conserved flanking regions of the ITS reads were trimmed with the q2-itsxpress plugin integrated into QIIME2 (58). Afterwards, ITS reads were corrected and filtered as recommended by the q2-itsxpress plugin tutorial (https://forum.qiime2.org/t/q2-itsxpress-a-tutorial-on-a-qiime-2-plugin-to-trim-its-sequences/5780). For the taxonomic classification of ITS data sets, we used the UNITE 7.2 database (59). As for the 16S data, we extracted the reference sequences from the databases and trained the Naïve Bayes classifier (57). However, we did not extract the target sequences of ITS primers but used the full reference sequences as suggested in the q2-feature-classifier tutorial (https://docs.qiime2.org/2018.6/tutorials/feature-classifier/).

Downstream analyses were conducted with the phyloseq, vegan, ampvis2, and ggplot2 R packages or custom R scripts (60–64). Samples with fewer than 1,000 reads for 16S and 200 reads for ITS were excluded from the resulting table. Moreover, taxonomically unassigned reads at the kingdom level and reads assigned to mitochondrial or other plant sequences were excluded from further analyses. A summary of the 16S and ITS data before and after filtering is available in Table S2. Before computing α diversity indices, the samples were rarefied to even depth. The α diversity indices of the samples were estimated from the observed number of features (i.e., richness) and as Shannon diversity metrics. The global significance of differences in microbial diversity among wheat genotypes in the axenic experiment and pairwise multiple comparisons between wheat genotypes was tested using a Kruskal-Wallis test (krus.test in R) and Conover’s test in the PMCMR R package, where we corrected the *P* values with the Holm correction method (65). In the soil experiment, two focal variables (wheat genotype and soil type, as well as the interaction of wheat genotype and soil type) were considered in the calculation of global significance of differences in microbial diversity, and this was tested by using the two-way ANOVA in R (aov function), where Tukey’s honest test was used as a *post hoc* test (TukeyHSD in R). Here, the diversity data were tested for a normal distribution (shapiro.test), and if the distribution was not normal, it was transformed in R (sqrt function).

After filtering and denoising, no fungal or bacterial features remained in the negative controls of DNA extraction and sterilization. The α diversity rarefaction plots for each sample confirmed that a sufficient depth of coverage of the 16S and ITS data sets was achieved for both individual seeds and seedlings. Further details about the amplification and sequencing are included in the supplemental material (Text S1).

To compare the compositions of communities and relative abundances of microbial taxa among wheat genotypes, the counts from the feature tables were normalized by the cumNorm function in the “metagenomeSeq” package (66). We computed the Jaccard, Bray-Curtis, and weighted and unweighted UniFrac distances to compare the structures of bacterial communities between samples. The Jaccard measure accounts for the absence/presence of taxa and the Bray-Curtis measure for both absence/presence and abundances, while the UniFrac metrics incorporate phylogenetic relatedness of bacterial communities into the calculation of distances. For fungal communities, we used only the Bray-Curtis and Jaccard metrics, as phylogeny-based metrics for the fungal data may lead to erroneous inference of phylogeny due to sequence length variation in ITS. The β diversity distance matrices were used for principal coordinate analysis (PCoA). A Kruskal-Wallis test and *post hoc* Conover’s test with false-discovery rate (FDR) correction for multiple comparisons were performed in the comparison of pairwise β diversities in different wheat genotypes in the axenic experiment. However, in the soil experiment, a two-way ANOVA and *post hoc* Tukey’s honest test were performed by considering two focal variables—soil type and wheat genotype—as well as their interactions. Permutational multivariate analysis of variance (PERMANOVA) based on the Bray-Curtis metrics was performed to test the significance of the effect of soil type and host genotype and their interactions in the microbial community composition (adonis function in the vegan package in R). For the detection of seed microbial communities, no parameter (including provenance) was explaining the variation. Also, only one population from each of wild wheat, landrace, and cultivar was included in the axenic and soil experiments. Therefore, we did not include “population/provenance” as a variable in the analysis.

### Data availability.

Scripts for preprocessing and downstream analysis of the data are available under https://github.com/ozkurt/wheat-microbiome, and raw MiSeq reads are available in NCBI under accession no. PRJNA667691.
