# Supplementary material for: Seed-Derived Microbial Colonization of Wild Emmer and Domesticated Bread Wheat (Triticum dicoccoides and T. aestivum) Seedlings Shows Pronounced Differences in Overall Diversity and Composition
Source: mBio. 2020 Nov 17;11(6):e02637-20. doi: 10.1128/mBio.02637-20 (PMC7683402; doi:10.1128/mBio.02637-20)
Supplement: FIG S5 [file mBio.02637-20-sf005.pdf]

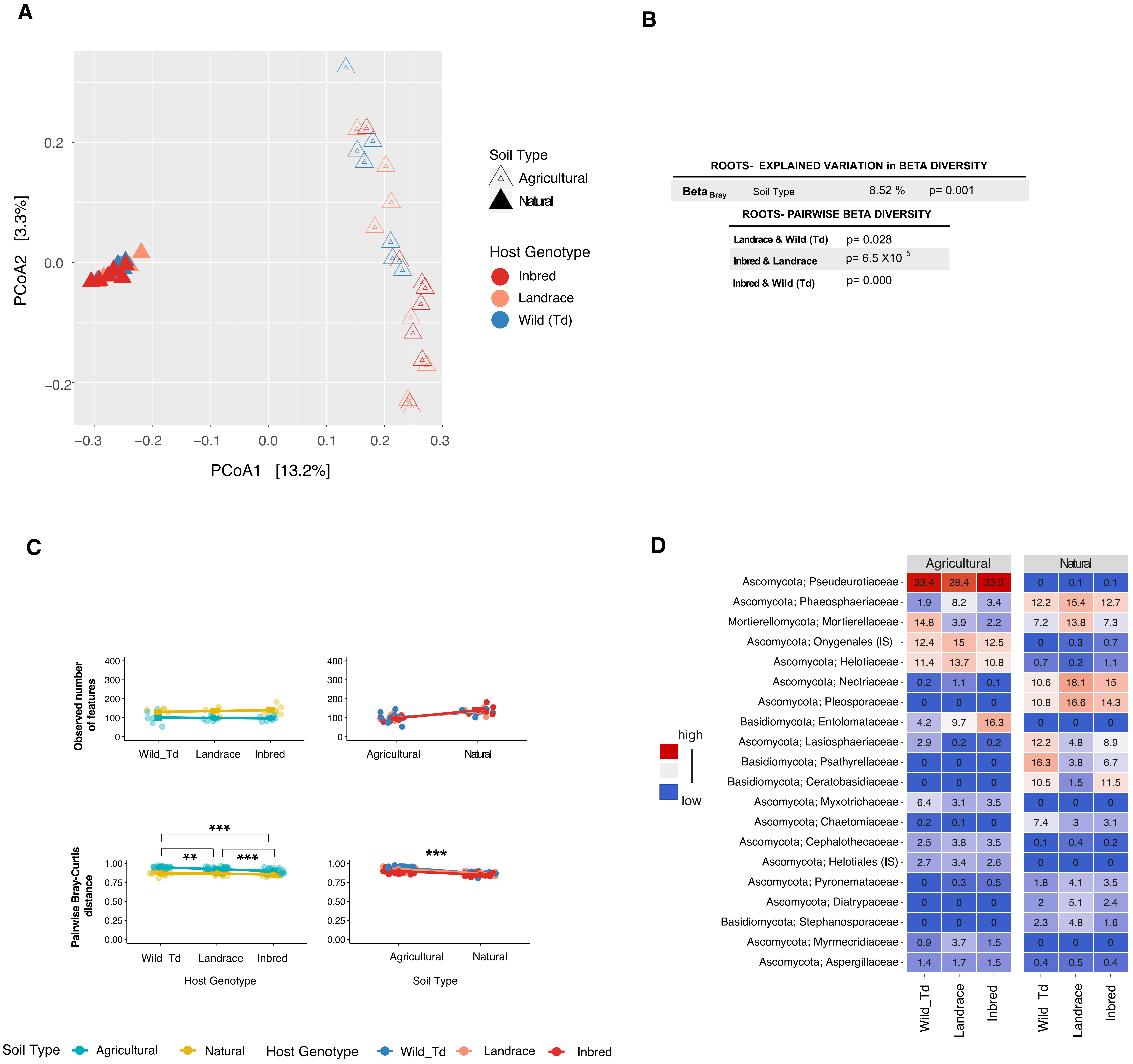
A
PCoA2 [3.3%]
0.2
0.0
−0.2
−0.3
−0.2
−0.1
0.0
0.1
0.2
0.3
PCoA1 [13.2%]
Soil Type
Agricultural
Natural
Host Genotype
Inbred
Landrace
Wild (Td)
B
ROOTS- EXPLAINED VARIATION in BETA DIVERSITY
Beta Bray
Soil Type
8.52 %
p= 0.001
ROOTS- PAIRWISE BETA DIVERSITY
Landrace & Wild (Td)
p= 0.028
Inbred & Landrace
p= 6.5 X10-5
Inbred & Wild (Td)
p= 0.000
C
Observed number of features
400
300
200
100
0
Wild_Td
Landrace
Inbred
Agricultural
Natural
Pairwise Bray-Curtis distance
1.00
0.75
0.50
0.25
0.00
***
**
***
Host Genotype
Soil Type
Soil Type
Agricultural
Natural
Host Genotype
Wild_Td
Landrace
Inbred
D
Agricultural
Natural
Ascomycota; Pseudeurotiaceae 33.4 28.4 33.9 0 0.1 0.1
Ascomycota; Phaeosphaeriaceae 1.9 8.2 3.4 12.2 15.4 12.7
Mortierellomycota; Mortierellaceae 14.8 3.9 2.2 7.2 13.8 7.3
Ascomycota; Onygenales (IS) 12.4 15 12.5 0 0.3 0.7
Ascomycota; Helotiaceae 11.4 13.7 10.8 0.7 0.2 1.1
Ascomycota; Nectriaceae 0.2 1.1 0.1 10.6 18.1 15
Ascomycota; Pleosporaceae 0 0 0 10.8 16.6 14.3
Basidiomycota; Entolomataceae 4.2 9.7 16.3 0 0 0
Ascomycota; Lasiosphaeriaceae 2.9 0.2 0.2 12.2 4.8 8.9
Basidiomycota; Psathyrellaceae 0 0 0 16.3 3.8 6.7
Basidiomycota; Ceratobasidiaceae 0 0 0 10.5 1.5 11.5
Ascomycota; Myxotrichaceae 6.4 3.1 3.5 0 0 0
Ascomycota; Chaetomiaceae 0.2 0.1 0 7.4 3 3.1
Ascomycota; Cephalothecaceae 2.5 3.8 3.5 0.1 0.4 0.2
Ascomycota; Helotiales (IS) 2.7 3.4 2.6 0 0 0
Ascomycota; Pyronemataceae 0 0.3 0.5 1.8 4.1 3.5
Ascomycota; Diatrypaceae 0 0 0 2 5.1 2.4
Basidiomycota; Stephanosporaceae 0 0 0 2.3 4.8 1.6
Ascomycota; Myrmecridiaceae 0.9 3.7 1.5 0 0 0
Ascomycota; Aspergillaceae 1.4 1.7 1.5 0.4 0.5 0.4
Wild_Td
Landrace
Inbred
Wild_Td
Landrace
Inbred
high
low
