# Supplementary material for: Seed-Derived Microbial Colonization of Wild Emmer and Domesticated Bread Wheat (Triticum dicoccoides and T. aestivum) Seedlings Shows Pronounced Differences in Overall Diversity and Composition
Source: mBio. 2020 Nov 17;11(6):e02637-20. doi: 10.1128/mBio.02637-20 (PMC7683402; doi:10.1128/mBio.02637-20)
Supplement: FIG S6 [file mBio.02637-20-sf006.pdf]

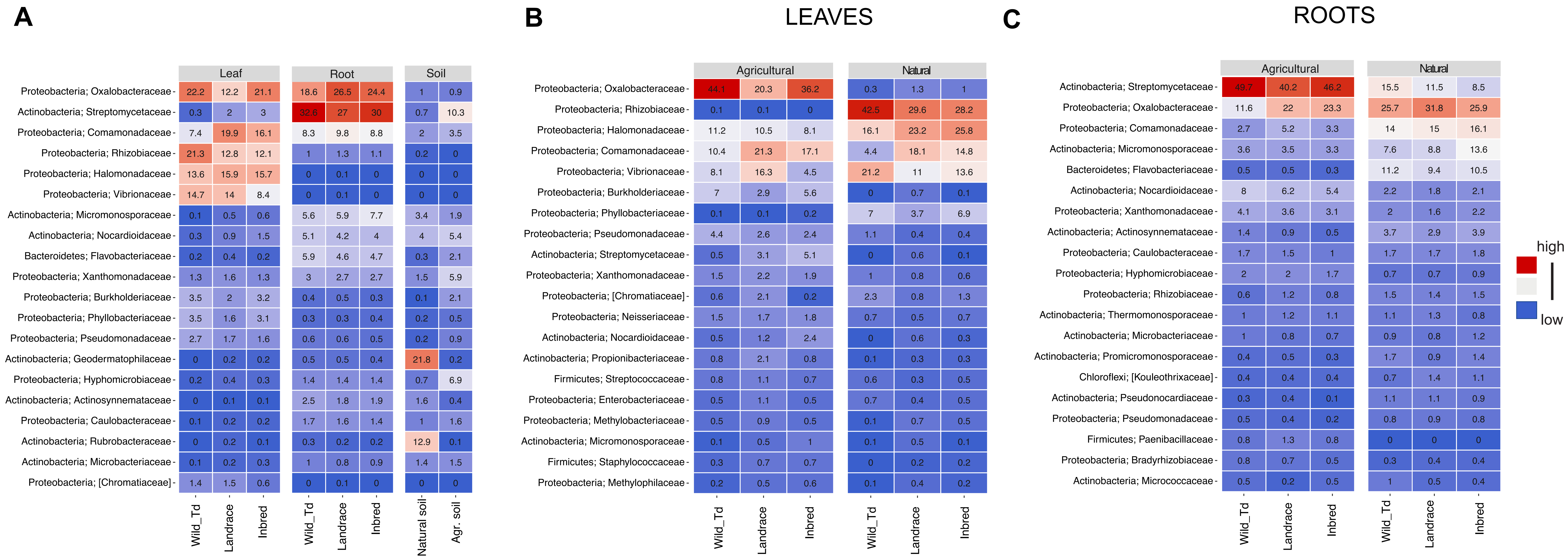
A
Leaf
Root
Soil
Proteobacteria; Oxalobacteraceae 22.2 12.2 21.1 18.6 26.5 24.4 1 0.9
Actinobacteria; Streptomycetaceae 0.3 2 3 32.6 27 30 0.7 10.3
Proteobacteria; Comamonadaceae 7.4 19.9 16.1 8.3 9.8 8.8 2 3.5
Proteobacteria; Rhizobiaceae 21.3 12.8 12.1 1 1.3 1.1 0.2 0
Proteobacteria; Halomonadaceae 13.6 15.9 15.7 0 0.1 0 0 0
Proteobacteria; Vibrionaceae 14.7 14 8.4 0 0.1 0 0 0
Actinobacteria; Micromonosporaceae 0.1 0.5 0.6 5.6 5.9 7.7 3.4 1.9
Actinobacteria; Nocardioidaceae 0.3 0.9 1.5 5.1 4.2 4 4 5.4
Bacteroidetes; Flavobacteriaceae 0.2 0.4 0.2 5.9 4.6 4.7 0.3 2.1
Proteobacteria; Xanthomonadaceae 1.3 1.6 1.3 3 2.7 2.7 1.5 5.9
Proteobacteria; Burkholderiaceae 3.5 2 3.2 0.4 0.5 0.3 0.1 2.1
Proteobacteria; Phyllobacteriaceae 3.5 1.6 3.1 0.3 0.3 0.4 0.2 0.5
Proteobacteria; Pseudomonadaceae 2.7 1.7 1.6 0.6 0.6 0.5 0.2 0.9
Actinobacteria; Geodermatophilaceae 0 0.2 0.2 0.5 0.5 0.4 21.8 0.2
Proteobacteria; Hyphomicrobiaceae 0.2 0.4 0.3 1.4 1.4 1.4 0.7 6.9
Actinobacteria; Actinosynnemataceae 0 0.1 0.1 2.5 1.8 1.9 1.6 0.4
Proteobacteria; Caulobacteraceae 0.1 0.2 0.2 1.7 1.6 1.4 1 1.6
Actinobacteria; Rubrobacteraceae 0 0.2 0.1 0.3 0.2 0.2 12.9 0.1
Actinobacteria; Microbacteriaceae 0.1 0.2 0.3 1 0.8 0.9 1.4 1.5
Proteobacteria; [Chromatiaceae] 1.4 1.5 0.6 0 0.1 0 0 0
Wild_Td Landrace Inbred Wild_Td Landrace Inbred Natural soil Agr. soil
B
LEAVES
Agricultural
Natural
Proteobacteria; Oxalobacteraceae 44.1 20.3 36.2 0.3 1.3 1
Proteobacteria; Rhizobiaceae 0.1 0.1 0 42.5 29.6 28.2
Proteobacteria; Halomonadaceae 11.2 10.5 8.1 16.1 23.2 25.8
Proteobacteria; Comamonadaceae 10.4 21.3 17.1 4.4 18.1 14.8
Proteobacteria; Vibrionaceae 8.1 16.3 4.5 21.2 11 13.6
Proteobacteria; Burkholderiaceae 7 2.9 5.6 0 0.7 0.1
Proteobacteria; Phyllobacteriaceae 0.1 0.1 0.2 7 3.7 6.9
Proteobacteria; Pseudomonadaceae 4.4 2.6 2.4 1.1 0.4 0.4
Actinobacteria; Streptomycetaceae 0.5 3.1 5.1 0 0.6 0.1
Proteobacteria; Xanthomonadaceae 1.5 2.2 1.9 1 0.8 0.6
Proteobacteria; [Chromatiaceae] 0.6 2.1 0.2 2.3 0.8 1.3
Proteobacteria; Neisseriaceae 1.5 1.7 1.8 0.7 0.5 0.7
Actinobacteria; Nocardioidaceae 0.5 1.2 2.4 0 0.6 0.3
Actinobacteria; Propionibacteriaceae 0.8 2.1 0.8 0.1 0.3 0.3
Firmicutes; Streptococcaceae 0.8 1.1 0.7 0.6 0.3 0.5
Proteobacteria; Enterobacteriaceae 0.5 1.1 0.5 0.7 0.4 0.5
Proteobacteria; Methylobacteriaceae 0.5 0.9 0.5 0.1 0.7 0.5
Actinobacteria; Micromonosporaceae 0.1 0.5 1 0.1 0.5 0.1
Firmicutes; Staphylococcaceae 0.3 0.7 0.7 0 0.2 0.2
Proteobacteria; Methylophilaceae 0.2 0.5 0.6 0.1 0.4 0.2
Wild_Td Landrace Inbred Wild_Td Landrace Inbred
C
ROOTS
Agricultural
Natural
Actinobacteria; Streptomycetaceae 49.7 40.2 46.2 15.5 11.5 8.5
Proteobacteria; Oxalobacteraceae 11.6 22 23.3 25.7 31.8 25.9
Proteobacteria; Comamonadaceae 2.7 5.2 3.3 14 15 16.1
Actinobacteria; Micromonosporaceae 3.6 3.5 3.3 7.6 8.8 13.6
Bacteroidetes; Flavobacteriaceae 0.5 0.5 0.3 11.2 9.4 10.5
Actinobacteria; Nocardioidaceae 8 6.2 5.4 2.2 1.8 2.1
Proteobacteria; Xanthomonadaceae 4.1 3.6 3.1 2 1.6 2.2
Actinobacteria; Actinosynnemataceae 1.4 0.9 0.5 3.7 2.9 3.9
Proteobacteria; Caulobacteraceae 1.7 1.5 1 1.7 1.7 1.8
Proteobacteria; Hyphomicrobiaceae 2 2 1.7 0.7 0.7 0.9
Proteobacteria; Rhizobiaceae 0.6 1.2 0.8 1.5 1.4 1.5
Actinobacteria; Thermomonosporaceae 1 1.2 1.1 1.1 1.3 0.8
Actinobacteria; Microbacteriaceae 1 0.8 0.7 0.9 0.8 1.2
Actinobacteria; Promicromonosporaceae 0.4 0.5 0.3 1.7 0.9 1.4
Chloroflexi; [Kouleothrixaceae] 0.4 0.4 0.4 0.7 1.4 1.1
Actinobacteria; Pseudonocardiaceae 0.3 0.4 0.1 1.1 1.1 0.9
Proteobacteria; Pseudomonadaceae 0.5 0.4 0.2 0.8 0.9 0.8
Firmicutes; Paenibacillaceae 0.8 1.3 0.8 0 0 0
Proteobacteria; Bradyrhizobiaceae 0.8 0.7 0.5 0.3 0.4 0.4
Actinobacteria; Micrococcaceae 0.5 0.2 0.5 1 0.5 0.4
Wild_Td Landrace Inbred Wild_Td Landrace Inbred
high
low
