# Supplementary material for: Seed-Derived Microbial Colonization of Wild Emmer and Domesticated Bread Wheat (Triticum dicoccoides and T. aestivum) Seedlings Shows Pronounced Differences in Overall Diversity and Composition
Source: mBio. 2020 Nov 17;11(6):e02637-20. doi: 10.1128/mBio.02637-20 (PMC7683402; doi:10.1128/mBio.02637-20)
Supplement: TEXT S1 [file mBio.02637-20-s0001.docx]

**Higher stochasticity of microbiota composition in seedlings of domesticated wheat compared to wild wheat**

Ezgi Özkurt, M. Amine Hassani, Uğur Sesiz, Sven Künzel, Tal Dagan, Hakan Özkan, Eva H. Stukenbrock

**Supplementary Methods**

**Origin of the wheat material**

Seed collections of *Triticum dicoccoides, Triticum boeoticum*and *Triticum urartu* were collected from different locations in South-East Turkey in previous years (2004, 2005, 2006) (Suppl Table 1). Seeds of *T. aestivum* from Turkey were collected by the farmer from a field in Kışlak, Antakya in 2017 (Suppl Table 1) and stored in a dry and cold room. We obtained these seeds in the same year from the farmer and we stored them at 4 °C until further usage. Seeds of *T. aestivum* from Germany (Benchmark cv.) were collected at the Kiel University experimental farm Hohenschulen, Schleswig-Holstein in 2017. All the seeds in the collections were stored at 4°C until further usage and processed all the seeds in the collections in 2018.

**Surface sterilization of seeds**

Seeds of *Triticum aestivum*, *Triticum dicoccoides*, *Triticum boeoticum* and *Triticum urartu* were washed with sterile miliQ water. Later, each seed was treated shortly with 0.1% TritonX and washed with water, then 80% EtOH and washed with water and lastly 1.2% Sodium Hypochlorite (NaClO) and finally washed with water three times. Wash-off water from some seeds were kept and sequenced. Some seeds were immediately processed for DNA extraction while others were grown in sterile jars. To ensure the efficiency of sterilization, we also pipetted the water from the last washing step into the R2A, dYT and YMS media to identify any potential bacterial or fungal contaminants. Furthermore, we used the negative control samples for PCR and sequencing. Confirming the efficiency oft he sterilization, we did not detect growth of any bacterial ot fungal colony in the media and no reads from the control remained in the analysis after filtering of the low quality reads.

**Axenic propagation of leaves and roots**

After sterilization, seeds were directly transferred into the plant minimal medium (PNM). 1L of PNM medium is composed 1 mL of of 500 mM Potassium Nitrate, 1mL of KH2PO4 stock solution (5g/ 100mL), 1 mL of K2HPO4 stock solution (2.5 g/100mL), 1mL of 2M Magnesium Sulfate (MgSO4 X 7 H2O), 1 mL of 200 mM Calcium Nitrate, 2.5 mL of Fe-EDTA and 1mL of Sodium Chloride stock solution (2.5 g/100 mL). The pH of the solution was adjusted to 6.2, and the medium was solidified by adding Gelrite. Finally, the medium was autoclaved at 121^o^C for 15 minutes. After autoclaving, 10 mL 1M filtered MES was added to the medium. A sample from the medium was processed for DNA extraction and microbial DNA amplification. No DNA amplification occurred, confirming the sterility of the growth medium. The medium was transferred into sterile jars and after it was solidified, sterilized seeds were transferred into the medium. Seeds were germinated in sterile jars in a climate chamber (Percival plant growth chambers, CLF PlantClimatics GmbH, Wertingen, Germany) with light period of 16/8 hours, temperature 15 ^o^C) for two weeks corresponding to the second leaf growth stage.

**DNA Extraction from seeds and seedling material**

We followed a similar method to the Phenol-Chloroform extraction method customized by Agler et al, 2016 [(1)](https://paperpile.com/c/Qt2IWU/1KR4). For DNA extractions, seed, leaf and root materials were processed as follows: Single seeds were transferred into 7mL tubes containing sterilized zirconium beads. Approximately six cm of leaves and six cm of multiple roots were used for DNA extraction. We crushed samples with Bertin Precellys Instrument (Bertin Instruments, Montigny-le-Bretonneux, France) pre-cooled with liquid nitrogen at 7500 rpm for 2 x 30 seconds with an in-between pause of 15 seconds. To the crushed samples, we added 0.6 mL of DNA extraction buffer (0.5% SDS, 50 mM TRIS buffer at pH 8, 200 mM NaCl, 2mM EDTA). Next, we added 50 µg/mL of Lyzozyme and 10 µg/mL of Proteinase K and incubated the samples for 45 min at 37^o^C. After incubation, samples were transferred into new tubes containing 0.1 mm zirconium and 0.5mm glass beads and beat-treated using Bertin Precellys (Bertin Instruments, Montigny-le-Bretonneux, France) at 6500 rpm for 2 x 30 seconds with a 15 second pause. 10 µg/mL RNAse was added to each sample before incubation at 37^o^C for 45 minutes. We next centrifuged the samples at 13000 rpm for 2 minutes to remove beads and non-homogenized tissue. The nucleic acids were cleaned up with phenol/chloroform/isoamyl alcohol (25:24:1) for three times, and chloroform/isoamyl alcohol (24:1) one time, then precipitated by adding 1/10th of the sample volume of 3 M sodium acetate and 2.5x volume 100% ethanol. Samples were then centrifuged at 4^o^C at 13000 rpm for 30 minutes. Afterwards, the pellet was washed with 70% ethanol. Finally, pellets were washed in 100 μL of sterile water and stored at -20 ^o^C until further usage. DNA extracted from seeds was treated with 1:1 with 20% Chelex-100 for 30 minutes to remove potential PCR inhibitors. Harvested leaves and roots were processed with the PowerSoil DNA kit (Mo Bio Laboratories, Heidelberg, Germany) according to the manufacturer’s instructions (See Material and Methods). Negative controls were processed along samples during DNA extraction and three randomly selected controls were sequenced for detection of any microbial contaminants. We validated the absence of contaminants as no reads remained in the analysis of controls after the filtering of low quality reads.

**DNA library preparation**

We prepared libraries for replicates of individual seed as well as leaf and root samples, respectively. In addition, we included three water negative controls of DNA extraction and three wash-off controls from the surface sterilization to the libraries.

We used the primers designed by Agler et al, 2016 (1). PCR amplification was performed in two steps (Suppl Table 1 and 2). For 16S, we used the interfering primer approach developed by Agler and co-workers for *Arabidopsis thaliana* (Suppl Table 3). However, we modified the primers according to the wheat genome [(2)](https://paperpile.com/c/Qt2IWU/3Ice). In the first step, the universal primer pairs B799F/ B1192R (targeting the V5/V6/V7 region) were used for bacterial sequences and for fungi the primer pair ITS1F/ITS2 (targeting ITS sequences) were used to amplify the targeted regions (Suppl Table 3). Interfering primers for the 16S region were added in this step. Triplicates of each reaction were performed for both first and second steps. First PCR was run for 15 and 20 cycles for 16S and ITS, respectively. After the first reactions, three replicates were combined. For the removal of primers and dNTPs prior to library preparation, 10 μL of each PCR product was treated with 0.5 μL Antarctic phosphatase and 0.5 μL Exonuclease I in 1.22 μL Antarctic phosphatase buffer (New England Biolabs GMBH, Frankfurt, Germany) at 37 °C for 30 minutes followed by 80 °C for 15 min. The product of the cleaned samples was used as a template for the second PCR in which unique barcodes for sequencing of 12bp length were added. Second PCRs were run for 20 and 15 cycles for 16S and ITS, respectively including in total 35 cycles for both 16S and ITS reactions.

**PCR conditions for the V5-7 amplification**

| **V5-V7** |  |  |  |  |
| --- | --- | --- | --- | --- |
| **1st PCR:** |  | **1st PCR conditions:** |  |  |
| 10 μL | Phusion Master Mix | 98°C | 30s |  |
| 1 μL | Forward Primer | **98°C** | **10s** |  |
| 1 μL | Reverse Primer | **55°C** | **45s** | **X15 cycles** |
| 2 μL | Interfering Forward Primer | **72°C** | **30s** |  |
| 2 μL | Interfering Reverse Primer | 72°C | 5m |  |
| 0.5 μL | DMSO |  |  |  |
| 3 μL | DNA |  |  |  |
| 0.5 μL | H2O |  |  |  |
|  |  |  |  |  |
| Clean-up with ExoI and Antartic Phosphatase | 10 μL of the 1st PCR product is cleaned-up | 37°C  80°C | 30m  15m |  |
|  |  |  |  |  |
| **2nd PCR:** |  | **2nd PCR conditions:** |  |  |
| 10 μL | Phusion Master Mix | 98°C | 30s |  |
| 1 μL | Forward Primer | **98°C** | 10s |  |
| 1 μL | Barcoded Reverse Primer | **55°C** | 45s | **X20 cycles** |
| 0.5 μL | DMSO | **72°C** | 30s |  |
| 3 μL | Clean-up product | 72°C | 5m |  |
| 4.5 μL | H2O |  |  |  |

**PCR conditions for the ITS1-ITS2 amplification**

| **ITS1F-ITS2** |  |  |  |  |
| --- | --- | --- | --- | --- |
| **1st PCR:** |  | **1st PCR conditions:** |  |  |
| 10 μL | Phusion Master Mix | 98°C | 30s |  |
| 1 μL | Forward Primer | **98°C** | **10s** |  |
| 1 μL | Reverse Primer | **55°C** | **30s** | **X20 cycles** |
| 0.5 μL | DMSO | **72°C** | **30s** |  |
| 1 μL | DNA | 72°C | 5m |  |
| 6.5 μL | H2O |  |  |  |
|  |  |  |  |  |
| Clean-up with ExoI and Antartic Phosphatase | 10 μL of the 1st PCR product is cleaned-up | 37°C  80°C | 30m  15m |  |
|  |  |  |  |  |
| **2nd PCR:** |  | **2nd PCR conditions:** |  |  |
| 10 μL | Phusion Master Mix | 98°C | 30s |  |
| 1 μL | Forward Primer | **98°C** | **10s** |  |
| 1 μL | Barcoded Reverse Primer | **55°C** | **30s** | **X15 cycles** |
| 0.5 μL | DMSO | **72°C** | **30s** |  |
| 1 μL | Clean-up product | 72°C | 5m |  |
| 6.5 μL | H2O |  |  |  |

Finally, PCR products of ITS and 16S amplifications were sub-pooled in equal concentrations where the concentration of each sample was estimated using the software of gel visualizer (BIO RAD, Image Lab^TM^ software 5.2.1). Subpools of 16S amplicons were run on the gel and bacterial DNA was cut out of the gel. The DNA concentration of each pooled sample were quantified using the Qubit 3.0 fluorometer (Thermo Fisher Scientific, Darmstadt, Germany). Then, we combined subpools in equal concentrations into final pools. Finally, we quantified the final concentration of the final pool with Qubit 3.0 fluorometer.

**Amplicon Sequencing**

The final DNA library spiked with 10% PhiX genomic DNA (Illumina, San Diego, CA)

was used for MiSeq sequencing (Illumina, San Diego, CA) using the MiSeq Reagent Kit v3 (Illumina, San Diego, CA). 0.5 μM of forward, reverse and index sequencing primers complementary to the linker/primer region of the concatenated primers were added together (sequence of primers are available in Suppl Table 3). Paired-end sequencing was performed for 600 cycles for 301 bp amplicon size according to the Illumina instructions (http://os.bioprotocol.org/attached/file/20171217/miseq%20denature%20dilute%20libraries%20guide%2015039740%2003.pdf).

**References:**

1. [Agler MT, Mari A, Dombrowski N, Hacquard S. New insights in host-associated microbial diversity with broad and accurate taxonomic resolution. bioRxiv [Internet]. 2016; Available from:](http://paperpile.com/b/Qt2IWU/1KR4) <https://www.biorxiv.org/content/early/2016/04/23/050005.abstract>

2. [Zimin AV, Puiu D, Hall R, Kingan S, Clavijo BJ, Salzberg SL. The first near-complete assembly of the hexaploid bread wheat genome, Triticum aestivum. Gigascience. 2017 Nov 1;6(11):1–7.](http://paperpile.com/b/Qt2IWU/3Ice)
